# Prognostic Value of the Baseline and Early Changes in Monocyte-to-Lymphocyte Ratio for Short-Term Mortality among Critically Ill Patients with Acute Kidney Injury

**DOI:** 10.3390/jcm12237353

**Published:** 2023-11-28

**Authors:** Xinyao Luo, Dingyuan Wan, Ruoxin Xia, Ruoxi Liao, Baihai Su

**Affiliations:** 1 Department of Nephrology, West China Hospital, Sichuan University, Chengdu 610041, China; luoxinyao@stu.scu.edu.cn; 2 Department of Intensive Care Medicine, West China Hospital, Sichuan University, Chengdu 610041, China; wan_dingyuan@stu.scu.edu.cn; 3 Department of Optometry and Visual Science, West China School of Medicine, Sichuan University, Chengdu 610041, China; xiaruoxin@stu.scu.edu.cn

**Keywords:** monocyte-to-lymphocyte ratio, restricted cubic splines, change, acute kidney injury, MIMIC-IV

## Abstract

(1) Background: Inflammation plays an important role in the onset and progression of acute kidney injury (AKI). Despite this, evidence regarding the prognostic effect of the monocyte-to-lymphocyte ratio (MLR), a novel systemic inflammation marker, among patients with AKI is scarce. This study sets out to investigate the prognostic potential of both baseline and early changes in MLR for short-term mortality among critically ill patients with AKI. (2) Method: Eligible patients with AKI from the Medical Information Mart for Intensive Care IV database were retrospectively analyzed. MLR cutoff values were determined using maximally selected rank statistics and tertiles. The clinical outcomes were 30-day and 90-day mortality in the intensive care unit. A restricted cubic splines model and Cox proportional hazards models were utilized to evaluate the association between the baseline MLR and short-term mortality. Then, the trends in MLR over time were compared between the 30-day survivors and non-survivors using a generalized additive mixed model (GAMM). (3) Result: A total of 15,986 patients were enrolled. Multivariable Cox regression analysis identified baseline MLR ≥ 0.48 as an independent risk factor predicting 30-day mortality (HR 1.33, 95%CI 1.24, 1.45, *p* < 0.001) and 90-day mortality (HR 1.34, 95%CI 1.23, 1.52, *p* < 0.001) after adjusting for potential confounders. Similar trends were observed for 30-day and 90-day mortality when tertiles were used to group patients. The restricted cubic splines model revealed a non-linear association between MLR and 30-day and 90-day mortality (both *p* for non-linear < 0.001, both *p* for overall < 0.001). The area under the curve of 0.64 for MLR was higher than that of monocytes (0.55) and lymphocytes (0.61). In the subgroup analyses, despite the noted significant interactions, the direction of the observed association between MLR and 30-day mortality was consistent across most prespecified subgroups, except for shock and black ethnicity. The GAMM results highlighted that, as time went on, MLR in the 30-day survival group consistently declined, whereas MLR in the non-survival group rose within 15 days post-ICU admission. The difference between the two groups persisted significantly even after adjusting for confounders (*p* = 0.006). (4) Conclusion: A higher baseline MLR was identified as an independent risk factor predicting 30-day and 90-day mortality. The early increase in MLR was associated with high 30-day mortality, suggesting that dynamic monitoring of MLR could potentially better predict survival in critically ill patients with AKI.

## 1. Introduction

Acute kidney injury (AKI), characterized by a rapid decline of kidney function, is a severe kidney disorder associated with a high morbidity and mortality rate [1,2]. Moreover, AKI stands as a prominent cause for an intensive care unit (ICU) admission, affecting 33–66% of adult critically ill patients [3]. In a meta-analysis including over 77 million hospitalized patients from 765 studies, the in-hospital mortality rate among AKI patients was approximately 21%, with a mortality rate of 42% in patients with AKI stage 3 [3].

Timely identification of patients at high risk of poor prognosis is pivotal for decreasing mortality [4]. Over the past decade, there has been a surge of observational studies devoted to identifying reliable clinical predictors of AKI mortality, such as neutrophil gelatinase-associated lipocalin [4,5], serum anion gap [6], and central venous pressure [7]. Unfortunately, the wide adoption of these biomarkers in clinical practice has been sluggish [8,9]. Multiple factors may contribute to this situation, including the recognized inertia of practitioners hindering the incorporation of novel findings into practice. Additionally, issues such as cost considerations and a lack of clinical evidence may play a role [8]. Therefore, there is a need to develop a simpler, more accessible, and cost-effective biomarker.

Dysregulated inflammation is critically involved in the pathogenesis and progression of AKI [10,11]. Many well-established inflammatory markers, including neutrophils [12], platelets [13], and platelet-to-lymphocyte ratio [14], have been associated with AKI prognosis. Monocyte-to-lymphocyte ratio (MLR), as a biomarker of inflammatory response [15,16], has also shown promising potential in predicting short-term outcomes in inflammation-related diseases, including sepsis [17,18], cancer [16,19], and cardiovascular diseases [20]. Recently, several studies suggested that MLR could independently predict the incidence of AKI in critically ill patients [21,22,23].

Given this evidence, it is plausible to speculate that MLR might impact the prognosis of AKI. However, there have been no epidemiological studies exploring the prognostic significance of MLR in critically ill patients with AKI. Therefore, aiming to capture the overall dynamics of the disease condition, we investigated the association between baseline and the early change within the first 15 days after ICU admission in MLR and all-cause mortality in these patients.

## 2. Materials and Methods

### 2.1. Data Source

The Medical Information Mart for Intensive Care IV ver. 2.0 (MIMIC-IV, version 2.0) is a large and openly accessible critical care database. It includes over 70,000 ICU admissions hospitalized at the Beth Israel Deaconess Medical Center (Boston, MA, USA) from 2008 to 2019. One author was approved to exploit data from the database after completing the National Institutes of Health’s web-based course known as Protecting Human Research Participants (LX, certification number 12059504).

The establishment of this database was approved by the institutional review boards of the Massachusetts Institute of Technology (MIT, Cambridge, MA, USA) and Beth Israel Deaconess Medical Center. All included patients were de-identified to protect their privacy. This study was a retrospective observational study, and it is reported based on the Strengthening the Reporting of Observational Studies in Epidemiology (STROBE) guideline.

### 2.2. Population Selection Criteria

Eligible patients with AKI were those who were older than 18 years old at admission and who had been hospitalized for more than 48 h. Patients were excluded from our study based on the following criteria: (1) >5% of their individual data were missing; (2) absence of data on monocyte and lymphocyte counts at the first admission; (3) baseline values exceeding the median ± 1.5 times the interquartile range; and (4) patients had a diagnosis of malignant neoplasms of the lymphatic and hematopoietic tissue. AKI was diagnosed according to the Kidney Disease: Improving Global Outcomes (KDIGO) guideline [24], which specifies changes in serum creatinine (SCr) levels and urine output. Stage 1 is defined as an increase ≥1.5 times baseline SCr within the prior 7 days or 0.3 mg/dl in SCr within 48 h, or urine output <0.5 mL/kg/h per 6 h. Stage 2 is characterized by an increase in SCr ≥2.0 times baseline or urine output <0.5 mL/kg/h per 12 h. Stage 3 is characterized by an increase in SCr ≥3.0 times baseline, SCr ≥4.0 mg/dl, initiation of renal replacement therapy (RRT), or urine output <0.5 mL/kg/h per 12 h. Eligibility was based on patients diagnosed with AKI according to KDIGO guidelines from 6 h before ICU admission up to 48 h after ICU admission. Baseline SCr refers to the lowest value within 7 days or 48 h prior to the AKI diagnosis.

### 2.3. Data Extraction

Patient data were extracted from MIMIC-IV using Structured Query Language (SQL) with PostgreSQL tools (version 15.1). The extracted data contained patient identifiers, clinical parameters, laboratory parameters, comorbidities, and scoring systems. The clinical parameters included age, race, gender, heart rate, respiratory rate, systolic blood pressure (SBP), diastolic blood pressure (DBP), mean arterial pressure (MAP), percutaneous oxygen saturation (SPO2), vasopressin used, ventilator used, and renal replacement therapy (RRT). The following laboratory parameters include glucose, chloride, anion gap, bicarbonate, lactate, blood urea nitrogen (BUN) levels, SCr levels, urine output, potassium, sodium, monocyte, lymphocyte, platelets, red blood cell (RBC), red cell distribution width (RDW), mean corpuscular volume (MCV), international normalized ratio (INR), and white blood cell (WBC). The MLR was defined as the ratio of the absolute monocyte count to the absolute lymphocyte count. Repeated measurements of MLR for each patient were performed during the first 15 days after ICU admission.

The following comorbidities were also extracted: hypertension, diabetes, coronary artery disease, chronic obstructive pulmonary disease (COPD), malignancy other than neoplasms of the lymphatic and hematopoietic tissue (abbreviated as malignancy in the following paragraphs), hematologic disease, atrial fibrillation, liver disease, shock, and sepsis based on the ninth or tenth revision of the International Classification of Diseases (ICD-9/10) code. The scoring systems, including the Sequential Organ Failure Assessment (SOFA) score and the Glasgow Coma Scale (GCS) score, were calculated for each patient. Only the data from the first admission to the ICU were taken into account for patients who had multiple admissions to the ICU. The first measured value within 6 h before ICU admission and within 48 h after ICU admission were used as the baseline data. The outcomes of our study were 30-day and 90-day mortality.

### 2.4. Statistical Analysis

Baseline characteristics were stratified by MLR cutoffs and are presented as frequency (percent) for categorical data and as mean (SD) or median (IQR) for continuous data. Comparisons between groups were made using the chi-square test for categorical variables. Analysis of variance, or the Mann–Whitney U test, was used for continuous variables. Survival curves were generated using the Kaplan–Meier method and compared using the log-rank test.

Cox proportional hazards models were constructed to test the associations between 30-day mortality and baseline covariates, with results expressed as hazard ratios (HR) with 95% confidence intervals (CIs). The group inclusion was based on cutoffs using maximally selected rank statistics and tertiles for MLR. Additionally, associations between the MLR and 90-day mortality were evaluated. To assess whether the MLR was independently associated with endpoints, we conducted a multivariable analysis using a stepwise selection modeling process.

Two multivariate models were constructed for both 30-day and 90-day all-cause mortality. The first tertile, or lower group, was treated as the reference group. In model 1, covariates were adjusted only for age, ethnicity, and sex; in model 2, we further adjusted for age, ethnicity, vasoactive use, RRT, GCS score, SOFA score, RDW, chloride, anion gap, potassium, MCV, BUN, SCr, urine output, INR, WBC, heart rate, temperature, SPO2, malignancy, shock, deficiency anemias, sepsis, coronary artery disease, liver disease, atrial fibrillation, and COPD.

To evaluate the potential non-linear relationships between the levels of MLR and 30-day and 90-day all-cause mortality, a restricted cubic spline was employed, aligning with Harrell’s recommendations [25] by placing four knots at the 5th, 35th, 65th, and 95th percentiles. The Wald test was utilized to assess the presence of nonlinearity in these relationships.

Time-dependent Receiver Operating Characteristic (ROC) analysis was used to assess the predictive ability of MLR, lymphocytes, monocytes, neutrophils, platelets, and SOFA for 30-day mortality. Through this analysis, the sensitivity and specificity of each index and the Area Under Curve (AUC) were computed. The optimal cut-off value of MLR was ascertained by the Youden index.

Subgroup analyses were conducted to evaluate potential variation in the efficacy of the MLR on different subgroups stratified by age, gender, ethnicity, heart rate, GCS score, SOFA, anion gap, potassium, SCr, BUN, RRT use, vasoactive drug use, and comorbidities (i.e., malignancy, shock, sepsis, deficiency anemias, coronary artery disease, COPD, and atrial fibrillation). To estimate multiplicative interactions, interaction terms were added based on the likelihood ratio test.

Furthermore, we investigated the association between early changes in MLR and 30-day mortality. A generalized additive mixed model (GAMM) was employed to evaluate the early changes in MLR over time between survivors and non-survivors, using both crude and fully adjusted models. The GAMM is particularly applied to analyze repeated measurements, especially when measurement intervals are irregular and some data are missing. Data analysis were conducted using R software version 4.2.2. Statistical significance was defined as a two-tailed *p* value less than 0.05.

## 3. Results

### 3.1. Subject Characteristics

**Figure 1:**
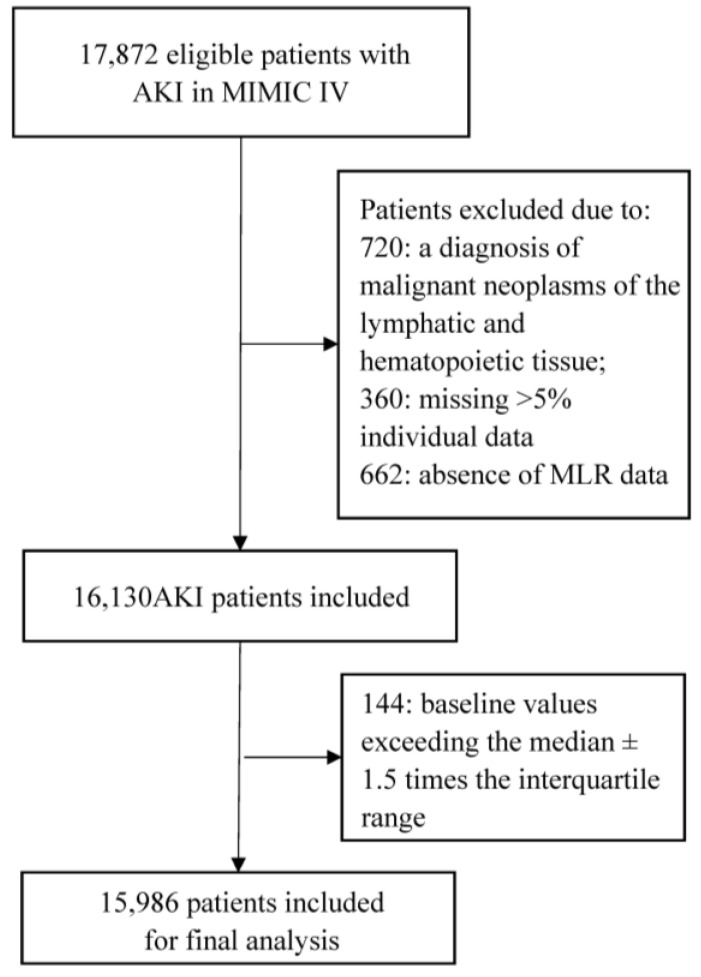
Flow chart of this study. Abbreviations: AKI, acute kidney injury; MIMIC IV, Medical Information Mart for Intensive Care IV.

The medical records of over 40,000 subjects who were admitted to the ICU at Beth Israel Deaconess Medical Center were initially obtained from the MIMIC-IV database. After applying the inclusion and exclusion criteria, a total of 15,986 eligible participants were included in our study (Figure 1). The mean age of participants was 67.0 years, comprising 9338 (58.4%) males and 6647 (41.6%) females.

The overall median MLR was 0.44 (IQR 0.25–0.77). Patients were divided into two groups based on the cut-off determined by maximally selected rank statistics (Supplementary Figure S1): the low-MLR group (MLR <0.48) consisted of 56.5% (*n* = 8577) of patients, while the high-MLR group (MLR ≥ 0.48) included 43.5% (n = 7409) of patients. The characteristics across MLR groupings are presented in Table 1. Patients in the high-MLR group (MLR ≥ 0.48) were more likely to be elderly, male, and have a comorbidity of COPD, malignancy, deficiency anemias, atrial fibrillation, shock, and sepsis; they also demonstrated a higher level of serum potassium, anion gap, glucose, BUN, SCr, platelets, neutrophils, WBC, AKI stages, and SOFA scores than patients in the low-MLR group (MLR < 0.48).

### 3.2. Association between Monocyte-to-Lymphocyte and 30-Day and 90-Day Outcomes

There were 2950 deaths (18.45%) within 30 days and 3824 deaths (23.92%) within 90 days during the follow-up period. In multivariate analysis, MLR stratified by maximally selected rank statistics was analyzed to determine whether MLR was independently associated with all-cause mortality (Table 2). In model I, adjusted for age, ethnicity, and gender, using the low MLRs (MLR < 0.48) as a reference, high MLRs (MLR ≥ 0.48) were independently associated with an increased risk of 30-day mortality and 90-day mortality (both *p* < 0.001). The HR (95%CI) for MLR were 2.11 (1.96–2.28) and 2.09 (1.96–2.23), respectively. In model II, after adjusting for age, ethnicity, vasoactive use, RRT use, GCS score, SOFA score, RDW, chloride, anion gap, MCV, BUN, SCr, urine output, INR, WBC, heart rate, temperature, SPO2, malignancy, shock, deficiency anemias, sepsis, coronary artery disease, liver disease, atrial fibrillation, and COPD, high MLR remained a significant predictor of all-cause mortality at 30 days and 90 days (HR, 95%CI: 1.33, 1.24–1.45; 1.34, 1.25–1.44, both *p* < 0.001).

Following the stratification of MLR into tertiles, compared with the referent group (MLR < 0.33), both medium (0.33 ≤ MLR < 0.66) and high MLRs (MLR ≥ 0.66) were independently associated with an increased risk of 30-day mortality (HR, 95%CI: 1.20, 1.07–1.33; 1.36, 1.23–1.52, both *p* < 0.001) after adjusting for the clinical confounders listed. A similar trend was observed for 90-day mortality, and the adjusted HRs (95%CI) for medium and high MLRs were 1.28 (1.16–1.40) and 1.43 (1.31–1.58), respectively.

**Figure 2:**
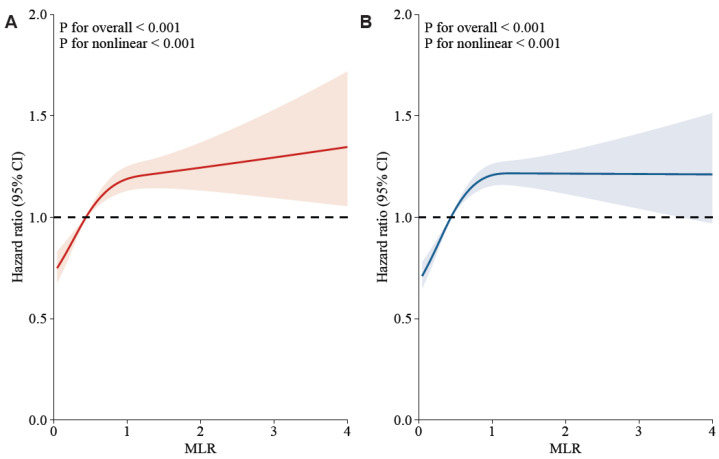
Potential nonlinearity for the levels of MLR with 30-day and 90-day mortality was measured by restricted cubic spline regression. The red and blue lines represented the estimated HR. The light red and blue areas represented the 95% CI. The MLR value of 4.8 was selected as the reference level. (**A**): MLR and 30-day mortality after adjusting the below confounders; (**B**): MLR and 90-day mortality after adjusting the below confounders. Adjusted confounders: age, ethnicity, vasoactive use, renal replacement therapy, Glasgow Coma Scale score, Sequential Organ Failure Assessment score, red cell distribution width, potassium, chloride, anion gap, mean corpuscular volume, blood urea nitrogen, serum creatinine, urine output, international normalized ratio, white blood cell, heart rate, temperature, percutaneous oxygen saturation, malignancy, shock, deficiency anemias, sepsis, coronary artery disease, liver disease, atrial fibrillation, and chronic obstructive pulmonary disease.

In Figure 2, a non-linear association of MLR with 30-day and 90-day mortality was demonstrated on a continuous scale with restricted cubic spline curved based on Cox proportional hazards models (both *p* for non-linear < 0.001, both *p* for overall < 0.001). A HR below 1 is observed when MLR is beneath 0.48, indicating mortality decreases as MLR increases. However, an elevated mortality risk is denoted by the HR increasing significantly with the MLR exceeding 0.48. Notably, the increasing trend of HR is relatively flat, with MLR exceeding 1.

### 3.3. ROC Curve Analysis and Kaplan–Meier Survival Curve Analysis

We plotted ROC curves to evaluate the predictive ability of six indicators, namely MLR, lymphocytes, monocytes, neutrophils, platelets, and the SOFA score, for 30-day mortality in patients with AKI. The detailed information is presented in Supplementary Figure S2 and Table S1. Notably, the AUC of MLR [0.64 (95% CI: 0.63–0.65)] outperformed those of lymphocytes [0.61 (95% CI: 0.60–0.62)], monocytes [0.55 (95% CI: 0.54–0.57)], neutrophils [0.57 (95% CI: 0.56–0.58)], and platelets [0.52 (95% CI: 0.51–0.54)]. However, it was inferior to the SOFA score [0.68 (95% CI: 0.67–0.69)]. Furthermore, an optimal MLR cut-off value of 0.48 was identified, yielding a sensitivity of 62.7% and a specificity of 58.7%. Intriguingly, the cut-off value derived from the ROC curve aligns closely with the cut-off determined by maximally selected rank statistics.

**Figure 3:**
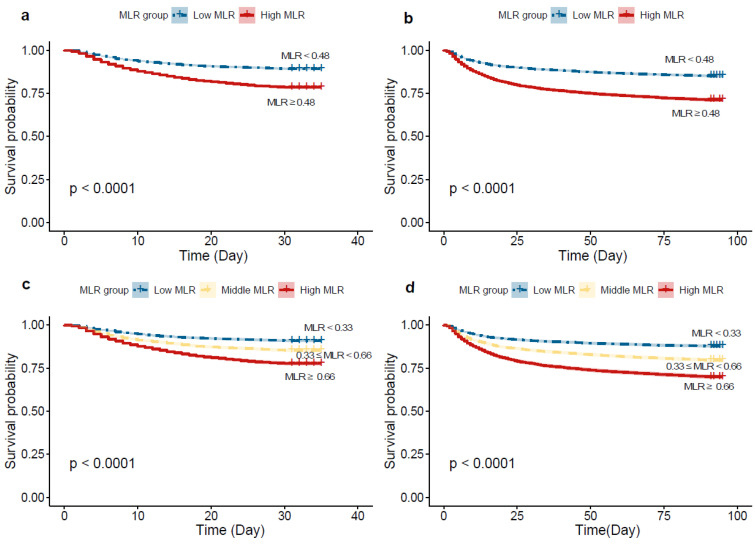
Kaplan–Meier curves of ICU 30-day and 90-day all-cause mortality for the low and high MLR groups. (**a**) grouping according to maximally selected rank statistics for 30-day mortality; (**b**) grouping according to maximally selected rank statistics for 90-day mortality; (**c**) grouping according to tertiles for 30-day mortality. (**d**). grouping according to tertiles for 90-day mortality.

In the Kaplan–Meier survival curve for 30-day mortality, as noted in Figure 3a,c, the cumulative survival rate is higher for the low-MLR group compared to the high-MLR group (log-rank test, chi-square = 277, *p* < 0.001). After stratifying MLR into tertiles, distinct survival outcomes were noted among the three groups over the 30-day follow-up period (log-rank test, chi-square = 282, *p* < 0.001). Similar results were observed in the 90-day mortality (Figure 3b,d).

### 3.4. Subgroup Analyses

In the subgroup analyses, the association between the MLR and the risk of 30-day mortality was similar in most strata (Supplementary Figure S3). Although significant interactions were observed for subgroups stratified by heart rate, GCS scores, SOFA scores, AKI stage, BUN, SCr, anion gap, RRT use, and comorbidities (i.e., shock, sepsis, coronary artery disease, and atrial fibrillation), the direction of association between the MLR and the risk of 30-day mortality remained stable in most strata, except for shock and ethnicity. Among the patients with shock, the association between MLR and 30-day mortality was not significant (HR, 95% CI: 1.11, 0.97–1.28). However, for patients without shock, MLR > 0.48 was significantly associated with higher 30-day mortality (HR, 95%CI: 1.52, 1.38–1.67). For patients of white and other ethnicities, higher MLR were associated with increased mortality (HR, 95% CI: 1.45, 1.31–1.60; HR, 95% CI: 1.42, 1.22–1.64), while no significant difference in mortality existed for black patients (HR, 95% CI: 1.23, 0.94–1.60).

### 3.5. Association between Early Changes in MLR and Mortality

**Figure 4:**
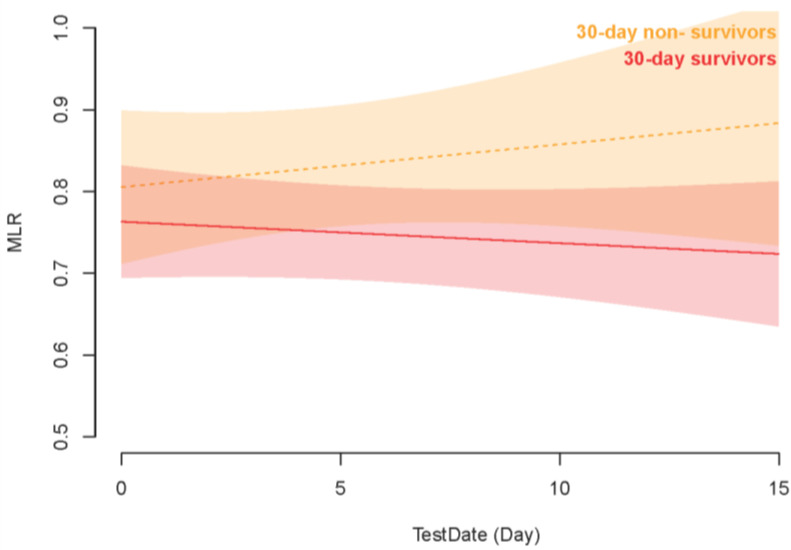
Association between the early changes (0–10 days) in MLR and 30-day mortality. Notes: A linear association between changes in MLR and 30-day mortality was found in a generalized additive mix model (GAMM). A smooth curve fitting graph illustrated the MLR based on the days after admission to the ICU. The red line represented the survivors. The yellow line represented the non-survivors. The pale red background represented 95% CIs in the survivors. The pale yellow background represented 95% CIs for the non-survivors.

We applied GAMM to analyze the early changes in MLR between 30-day survivors and non-survivors after adjusting for confounders. The GAMM result indicated that, as time went on, MLR decreased in the 30-day survival group, whereas in the non-survival group, the trend was reversed and MLR consistently increased within 15 days after ICU admission (Figure 4). Furthermore, as shown in Table 3, we further explored the early longitudinal changes of MLR for predicting 30-day mortality in patients with AKI. Notably, at a specific time point after ICU admission, the MLR in the survival group was significantly lower compared to the non-survival group (β = −0.079, *p* = 0.006), even after accounting for the listed clinical confounders. Moreover, as time progressed after ICU admission, MLR tended to increase (β = 0.252, *p* = 0.029) for all patients in this study, regardless of their survival status.

## 4. Discussion

The present study explored the baseline and dynamic changes in MLR for predicting short-term mortality in critically ill patients with AKI. In multivariate analysis, we found a significant independent association between elevated MLR and both 30-day and 90-day mortality, even after the adjustment for established confounding factors. The restricted cubic splines analysis elucidated a non-linear relationship between MLR and mortality at both 30 and 90 days. Comparison by AUC values showed higher accuracy of MLR (0.64) than monocyte (0.55) or lymphocyte (0.61) alone. Moreover, the GAMM result highlighted that there were significant differences in MLR during the first 15 days after ICU admission between the survivors and non-survivors. The association remained stable after adjusting for potential confounders. Therefore, dynamic monitoring of MLR may aid in identifying patients with worse survival probabilities.

AKI is not only characterized by immediate renal dysfunction but is also associated with a pronounced inflammatory response [1,10]. Accumulating evidence has indicated that various indicators of inflammation, including platelets, lymphocytes, monocytes, and neutrophils, are intricately involved in the progression of AKI [13,14,26,27]. Monocyte and lymphocyte levels, both individually and as a ratio, have been the subject of much research attention in relation to AKI. Monocytes migrate into the injured kidney and differentiate into macrophages, further infiltrating tissue and aggravating ischemic murine ischemic renal injury by secreting pro-inflammatory cytokines interleukin (IL)-6, tumor necrosis factor-α, and IL-1β [28,29,30]. Another experimental study by Guo et al. found that decreased expression of the gene AFM could lead to increased monocyte infiltration, thereby promoting renal inflammation and injury in murine models of sepsis-associated AKI [31]. Furthermore, it has been demonstrated that regulatory T lymphocytes (Treg) could exert protective effects in AKI, reducing inflammation and promoting tissue repair through the anti-inflammatory cytokine IL-10-mediated suppression of the innate system [32]. Jager et al. have demonstrated that low absolute lymphocyte counts could be a better predictor of bacteremia than conventional infection markers in the emergency care unit [33].

Taken together, the alteration in absolute levels of monocytes and lymphocytes could potentially reflect the balance between pro-inflammatory and anti-inflammatory states in the AKI setting. An elevated monocyte level might indicate an ongoing, active inflammatory process [34]. Concurrently, an increase in lymphocyte apoptosis might lead to fewer Treg cells available, which might signify an impaired anti-inflammatory response, thereby exacerbating the renal injury [35]. Hence, it is plausible to hypothesize that the MLR, by capturing both aspects, may serve as a more potent indicator in AKI populations than the assessment of monocyte or lymphocyte counts independently.

Recently, several studies have reported that MLR could predict AKI incidence. In a retrospective study enrolling 1500 ICU patients, Jiang et al. reported that the risk value of MLR for AKI occurrence was nearly three times higher than the neutrophil-to-lymphocyte ratio (OR = 3.904, 95% CI: 1.623–9.391 vs. OR = 1.161, 95% CI: 1.135–1.187, *p* < 0.001) [21]. The areas under the receiver operating characteristic curve for MLR in predicting AKI incidence were 0.899 (95% CI: 0.881–0.917) [21]. Another study conducted by the same research group revealed that MLR at admission was associated with an increased AKI risk, with an OR of 8.27 (95%CI: 4.23, 16.17, *p* < 0.001) in patients with acute hemorrhagic stroke [36]. However, no clinical study has reported the prognostic value of MLR for all-cause mortality, specifically in AKI populations, thus far. It has been recognized that a high MLR is associated with a short-term prognosis in various critically ill patients [23,37,38,39]. Recent research by Yang et al. revealed a significant association between a high MLR and a hazard ratio (HR) of 2.518 for all-cause mortality in patients undergoing peritoneal dialysis [37]. Moreover, Fen et al. have suggested that high MLR was associated with a higher in-hospital mortality (HR, 2.825, 95% CI: 1.058, 7.545) in acute ischemic stroke patients, whereas high NLR was associated with a risk of in-hospital mortality of 1.086 (95% CI: 1.022, 1.151) [23]. The prognostic value of MLR for all-cause mortality has also been substantiated in patients undergoing hemodialysis (HR, 95%CI: 2.518; 1.020–6.214, *p* = 0.0045) [38]. In a retrospective study with 461 patients, Muresan et al. observed that baseline MLR has a potent predictive capacity for all-cause 30-day mortality in end-stage renal disease patients [39].

In the present study, the high MLR group exhibited higher baseline age, blood pressure, heart rate, respiratory rate, and more severe comorbidities, such as sepsis, shock, atrial fibrillation, liver disease, malignancy, and COPD. Nevertheless, even after adjusting for these factors, MLR remained significantly associated with 30-day and 90-day mortality. This suggested that high MLR could independently predict short-term prognosis in critically ill patients with AKI, regardless of other confounding factors, which is also supported by the results from the Kaplan–Meier curve. Furthermore, the ROC analysis confirmed that MLR serves as a more robust indicator for AKI compared to the evaluation of monocyte or lymphocyte counts individually.

In the subgroup analysis, significant interactions were observed for subgroups stratified by GCS scores, SOFA scores, heart rate, temperature, anion gap, BUN, SCr, MCV, chloride, and comorbidities including shock, sepsis, coronary artery disease, and atrial fibrillation. However, association between MLR and 30-day mortality remained stable, except in cases of shock. This suggested that the influence of clinical heterogeneity on the prognostic effect of MLR was relatively small, reinforcing its potential utility as a biomarker in critically ill patients with AKI. Nevertheless, in patients with shock, we observed that MLR failed to serve as a predictive factor for prognosis. We speculated that patients with shock often present with multi-organ dysfunction and immune dysregulation, which may overshadow the prognostic significance of MLR due to the complexity of clinical factors and the overall severity of the patient’s condition. This observation aligns with a retrospective study by Liberski et al., which revealed that MLR failed to predict ICU mortality in patients with septic shock (HR, 95%CI: 0.96; 0.62–1.47; *p* = 0.84) [40]. However, another study conducted by Hsu et al. involving 93 patients found that a high lymphocyte-to-monocyte ratio (>0.89) was an independent factor for mortality in cirrhotic patients with septic shock (adjusted HR = 0.54, *p* = 0.01) [41]. Therefore, caution should be exercised about the potential impact of shock when evaluating the relationship between MLR and mortality in critically ill patients with AKI.

Furthermore, we utilized the GAMM to explore the association between early MLR and 30-day mortality in ICU patients. Even after adjusting for potential confounders, the differences in MLR between survivors and non-survivors were found to persist within the first 15 days following ICU admission, further strengthening the evidence for the role of MLR as a prognostic marker in critically ill patients with AKI. The increasing MLR in non-survivors might suggest a more severe inflammatory response, while in survivors, MLR consistently declined, leading us to hypothesize that the inflammatory state in these patients gradually subsided. Taken together, MLR could be obtained through routine blood tests, making it more cost-effective and convenient for clinical use. In addition, monitoring the dynamic alteration in MLR could potentially provide more timely prognostic information, thereby guiding intervention and risk stratification. It is important to note that while the MLR is a valuable prognostic biomarker, it does not play a role in preventing the development of AKI. Instead, its primary utility lies in its ability to predict prognosis and assist in assessing the severity and potential outcomes of the disease.

Our study is the first, to the best of our knowledge, to investigate the association between MLR and all-cause mortality in AKI. Moreover, the diversity of our study population enhances the generalizability of our findings. Despite these strengths, several limitations should be acknowledged. First, the single-center, retrospective nature of our study might have introduced selection bias. Future multi-center, prospective studies will be valuable in validating our findings. Second, despite our best efforts to adjust for known confounding factors using multivariate analysis, residual confounding by unknown factors might exist. Third, the relationship between early changes in MLR and mortality does not imply causality, and it remains uncertain whether adjusting MLR could impact short-term survival among patients with AKI. Therefore, a longitudinal study design with repeated measures of MLR may be valuable in delineating this relationship further. Finally, the prognostic value of MLR should be further confirmed, and the cutoff value should be established in one cohort and tested in another.

## 5. Conclusions

MLR stands as an independent predictor of 30-day and 90-day all-cause mortality in critically ill patients with AKI. Moreover, the early changes in MLR were associated with higher 30-day mortality in AKI patients. Due to its cost-effectiveness and availability, MLR holds significant potential as a clinical biomarker. Future studies, particularly large-scale prospective studies, are needed to verify its clinical value.

## Figures and Tables

**Table 1:** Baseline characteristics of this study population.

Characteristic	Monocyte-to-Lymphocyte Ratio			*p* Value
	All Patients	<0.48	≥0.48	
MLR	0.44 [0.25, 0.77]	0.26 [0.18, 0.36]	0.80 [0.61, 1.19]	<0.001
Age, years	67.56 (15.83)	67.03 (15.52)	68.17 (16.16)	<0.001
Sex, *n* (%)				0.953
Female	6648 (41.6)	3585 (41.8)	3063 (41.3)	
Ethnicity				0.001
White, *n* (%)	10,820 (67.7)	5784 (67.4)	5036 (68.0)	
Black, *n* (%)	1381 (8.6)	807 (9.4)	574 (7.7)	
Other, *n* (%)	3785 (23.7)	1986 (23.2)	1799 (24.3)	
SBP, mmHg	123.1 (24.8)	122.3 (24.4)	124.0 (25.3)	0.002
DBP, mmHg	67.6 (18.2)	66.5 (17.5)	69.0 (18.9)	<0.001
Heart rate, beats/minute	86.0 [76.0, 100.0]	82.0 [74.0, 95.0]	86.0 [76.0, 100.0]	<0.001
Respiratory rate, /minute	19.3 (6.0)	18.3 (5.7)	20.4 (6.3)	<0.001
Temperature, °C	36.64 (0.85)	36.54 (0.84)	36.75 (0.85)	<0.001
SpO2, %	98.00 [96.00, 100.00]	99.00 [96.00, 100.00]	98.00 [95.00, 100.00]	<0.001
Vasoactive use (%)	7774 (48.6)	4339 (50.6)	3435 (46.4)	<0.001
Ventilator use (%)	8681 (54.3)	4770 (55.6)	3911 (52.8)	0.006
Comorbidities				
Hypertension, *n* (%)	9365 (58.6)	5089 (59.3)	4276 (57.7)	0.040
Diabetes, *n* (%)	5253 (32.9)	2946 (34.3)	2307 (31.1)	<0.001
Coronary artery disease, *n* (%)	5969 (37.3)	3736 (43.6)	2233 (30.1)	<0.001
COPD, *n* (%)	1860 (11.6)	836 (9.7)	1024 (13.8)	<0.001
Malignancy, *n* (%)	1636 (10.2)	589 (6.9)	1047 (14.1)	<0.001
Liver disease, *n* (%)	2413 (15.1)	965 (11.3)	1448 (19.5)	<0.001
Deficiency anemias, *n* (%)	8097 (50.7)	4280 (49.9)	3817 (51.5)	0.043
Atrial fibrillation (%)	2940 (18.4)	1405 (16.4)	1535 (20.7)	<0.001
Shock (%)	2474 (15.5)	946 (11.0)	1528 (20.6)	<0.001
Sepsis (%)	9921 (62.1)	4746 (55.3)	5175 (69.8)	<0.001
Laboratory parameters				
Hematocrit, %	33.30 [28.10, 38.80]	32.60 [27.70, 38.40]	34.00 [28.70, 39.20]	<0.001
Hemoglobin, g/dL	10.90 [9.20, 12.80]	10.80 [9.10, 12.70]	11.10 [9.30, 12.90]	<0.001
Platelet count, 10^9^/L	196.00 [140.00, 267.00]	187.00 [135.00, 257.00]	205.00 [148.00, 278.00]	<0.001
WBC, 10^9^/L	11.60 [8.40, 15.90]	10.50 [7.80, 14.20]	13.00 [9.60, 17.90]	<0.001
RDW, %	14.30 [13.30, 15.80]	14.00 [13.20, 15.30]	14.60 [13.50, 16.30]	<0.001
MCV, fL	92.00 [88.00, 96.00]	91.00 [87.00, 95.00]	92.00 [88.00, 97.00]	<0.001
INR	1.30 [1.10, 1.50]	1.30 [1.10, 1.50]	1.30 [1.10, 1.60]	<0.001
Serum creatinine, mg/dL	1.00 [0.80, 1.60]	1.00 [0.80, 1.40]	1.10 [0.80, 1.80]	<0.001
BUN, mg/dL	20.00 [14.00, 33.00]	18.00 [13.00, 28.00]	23.00 [16.00, 39.00]	<0.001
Glucose, mg/dL	130.00 [107.00, 167.00]	126.00 [106.00, 158.00]	136.00 [110.00, 177.00]	<0.001
Serum sodium, mmol/L	139.00 [136.00, 141.00]	139.00 [136.00, 141.00]	138.00 [135.00, 141.00]	<0.001
Serum chloride, mmol/L	104.00 [99.00, 108.00]	105.00 [101.00, 109.00]	102.00 [98.00, 106.00]	<0.001
Serum bicarbonate, mmol/L	23.00 [20.00, 25.00]	23.00 [21.00, 25.00]	23.00 [20.00, 25.00]	<0.001
Anion gap, mmol/L	15.00 [12.00, 18.00]	14.00 [12.00, 17.00]	16.00 [13.00, 19.00]	<0.001
Serum potassium, mmol/L	4.20 [3.80, 4.70]	4.20 [3.80, 4.60]	4.30 [3.80, 4.80]	<0.001
Scoring systems				
GCS	14.00 [10.00, 15.00]	14.00 [10.00, 15.00]	13.00 [9.00, 15.00]	<0.001
SOFA	5.00 [3.00, 8.00]	5.00 [3.00, 7.00]	6.00 [3.00, 9.00]	<0.001
Renal function				
Urine output, mL/24 h	1802 (1205.03)	1889.77 (1145.03)	1686.52 (1270.25)	<0.001
AKI KDIGO stage, *n* (%)				<0.001
Stage-1	4053 (25.4)	2477 (28.9)	1576 (21.3)	
Stage-2	8034 (50.3)	4464 (52.0)	3570 (48.2)	
Stage-3	3899 (24.4)	1636 (19.1)	2263 (30.5)	
Renal replacement therapy, *n* (%)	849 (5.3)	335 (3.9)	514 (6.9)	<0.001

Abbreviations: SBP Systolic blood pressure, DBP Diastolic blood pressure, SpO2 percutaneous oxygen saturation, COPD chronic obstructive pulmonary disease, WBC white blood cell, RDW red cell distribution width, MCV mean corpuscular volume, INR international normalized ratio, BUN Blood urea nitrogen, GCS Glasgow Coma Scale, SOFA Sequential Organ Failure Assessment, KDIGO Kidney Disease: Improving Global Outcomes. Normally distributed data are presented as the mean (SD) (analysis of variance); non-normally distributed data are presented as the median (IQR) (nonparametric Wilcoxon test); and categorical variables are presented as *n* (%) (chi-square test).

**Table 2:** HRs (95% CIs) for all-cause mortality across groups of monocyte-to-lymphocyte ratios.

Monocyte-to-Lymphocyte Ratio	No. of Patients/Deaths	Model 1		Model 2	
		HR (95% CIs)	*p* Value	HR (95% CIs)	*p* Value
30-Day mortality					
Fitted groups					
<0.48	8577/1076	1.00		1.00	
≥0.48	7409/1874	2.11 (1.96–2.28)	<0.001	1.33 (1.24–1.45)	<0.001
Tertiles					
<0.33	5329/568	1.00		1.00	
0.33–0.66	5328/946	1.65 (1.48–1.85)	<0.001	1.20 (1.07–1.33)	0.007
≥0.66	5329/1436	2.55 (2.30–2.83)	<0.001	1.36 (1.23–1.52)	<0.001
90-Day mortality					
Fitted groups					
<0.48	8577/1426	1.00		1.00	
≥0.48	7409/2398	2.09 (1.96–2.23)	<0.001	1.34 (1.25–1.44)	<0.001
Tertiles					
<0.33	5329/729	1.00		1.00	
0.33–0.66	5328/1269	1.81 (1.65–1.98)	<0.001	1.28 (1.16–1.40)	<0.001
≥0.66	5329/1826	2.73 (2.51–2.98)	<0.001	1.44 (1.31–1.58)	<0.001

Models 1 and 2 were derived from Cox proportional hazards regression models; Model 1 covariates were adjusted for age, ethnicity, and sex; Model 2 covariates were adjusted for age, ethnicity, vasoactive use, renal replacement therapy, Glasgow Coma Scale score, Sequential Organ Failure Assessment score, red cell distribution width, potassium, chloride, anion gap, mean corpuscular volume, blood urea nitrogen, serum creatinine, urine output, international normalized ratio, white blood cell, heart rate, temperature, percutaneous oxygen saturation, malignancy, shock, deficiency anemias, sepsis, coronary artery disease, liver disease, atrial fibrillation, and chronic obstructive pulmonary disease.

**Table 3:** Relationship between changes (0–15 days) MLR and 30-day mortality in critically ill patients with AKI derived from a generalized additive mixed model (GAMM).

	Unadjusted β (95% CI)	*p*-Value	Adjusted Model I β (95% CI)	*p*-Value	Adjusted Model II β (95% CI)	*p*-Value
30-day mortality	−0.176 (−0.230, −0.123)	<0.001	−0.146 (−0.201, −0.091)	<0.001	−0.079 (−0.137, −0.029)	0.006
Day	0.205 (0.195, 0.214)	<0.001	0.119 (0.091, 0.146)	<0.001	0.252 (0.024, 0.480)	0.029

CI, confidence interval; 30-day mortality, the difference of MLR between 30-day survivors and non-survivor at a specific timepoint; Day, the mean of the increasing of MLR over time (daily) for all patients. Model 1 covariates were adjusted for age, ethnicity, and sex; Model 2 covariates were adjusted for age, ethnicity, vasoactive use, renal replacement therapy, Glasgow Coma Scale score, Sequential Organ Failure Assessment score, red cell distribution width, potassium, chloride, anion gap, mean corpuscular volume, blood urea nitrogen, serum creatinine, urine output, international normalized ratio, white blood cell, heart rate, temperature, percutaneous oxygen saturation, malignancy, shock, deficiency anemias, sepsis, coronary artery disease, liver disease, atrial fibrillation, and chronic obstructive pulmonary disease.

## Data Availability

The data for this study can be found at https://mimic.mit.edu/, accessed on 29 September 2023.

## Supplementary Materials

The following supporting information can be downloaded at: https://www.mdpi.com/article/10.3390/jcm12237353/s1. Figure S1: The optimal cut-point of MLR Determined by Maximally Selected Rank Statistics. Figure S2. ROC curves of MLR correlate for predicting 30-day mortality, MLR, monocyte to lymphocyte ratio; SOFA, sequential organ failure assessment. Figure S3. Subgroup analysis of the associations between 30-day all-cause mortality and the lymphocyte-to-lymphocyte ratio.
