# Supplementary material for: Prognostic Value of the Baseline and Early Changes in Monocyte-to-Lymphocyte Ratio for Short-Term Mortality among Critically Ill Patients with Acute Kidney Injury
Source: J Clin Med. 2023 Nov 28;12(23):7353. doi: 10.3390/jcm12237353 (PMC10707087; doi:10.3390/jcm12237353)
Supplement: Supplementary file 1 [file jcm-12-07353-s001.zip › jcm-2655596-supplementary.pdf]

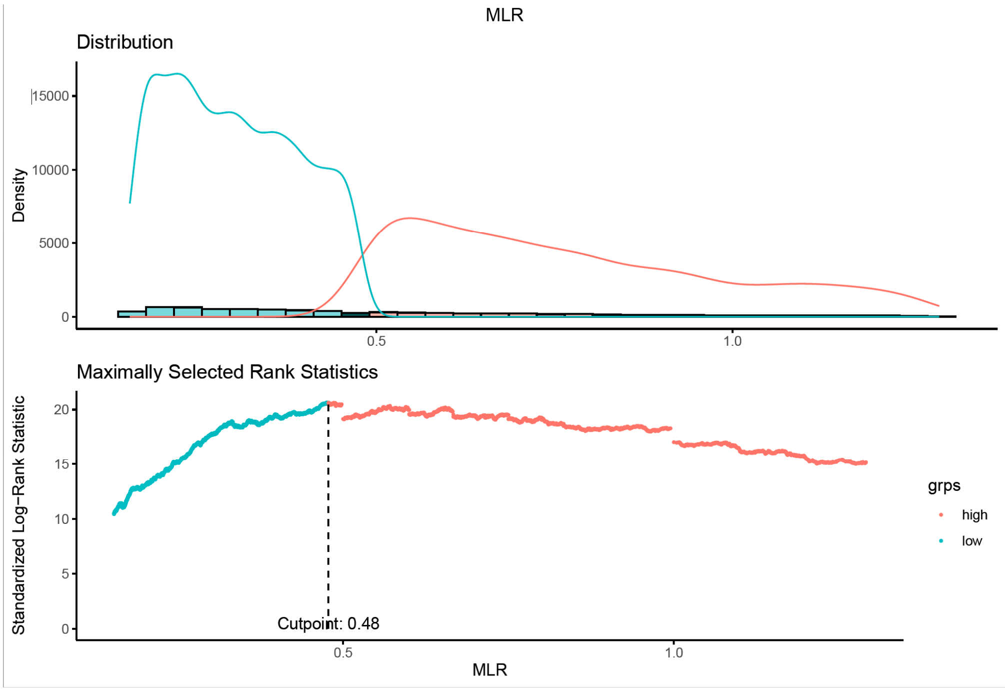

Figure S1. The optimal cut-point of MLR Determined by Maximally Selected Rank Statistics.

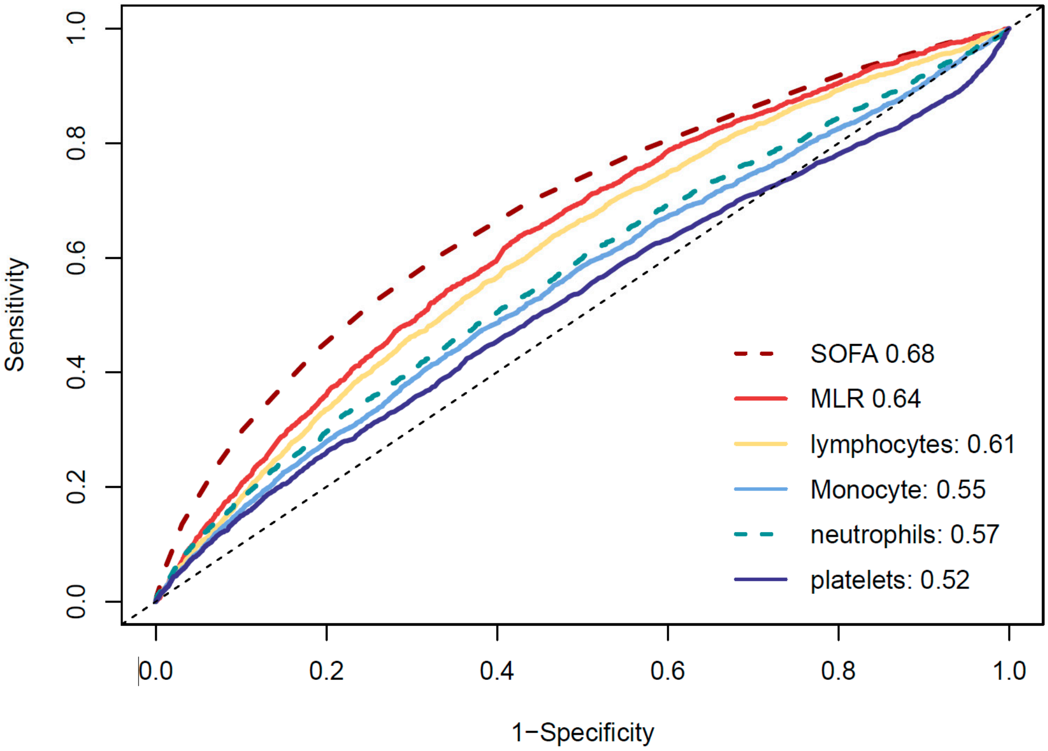

Figure S2. ROC curves of MLR correlate for predicting 30-day mortality, MLR, monocyte to lymphocyte ratio; SOFA, sequential organ failure assessment.

Variable	MLR<=0.48	MLR>0.48	HR (95% CI)	P for interaction
Overall	8577	7409	1.41 (1.31 to 1.53)	
Age				0.324
<=65	3504	2850	1.39 (1.21 to 1.60)	
>65	5073	4559	1.43 (1.30 to 1.57)	
Ethnicity				0.539
Black	807	574	1.23 (0.94 to 1.60)	
White	1986	1799	1.45 (1.31 to 1.60)	
Other	5784	5036	1.42 (1.22 to 1.64)	
Heart rate				0.001
<=100	6934	5094	1.49 (1.35 to 1.64)	
>100	1642	2310	1.22 (1.06 to 1.39)	
GCS score				<0.001
<=8	1551	1612	1.32 (1.17 to 1.50)	
8~12	5965	4513	1.30 (1.08 to 1.56)	
>12	1061	1282	1.56 (1.38 to 1.76)	
SOFA score				<0.001
<=6	5704	4106	1.57 (1.39 to 1.78)	
>6	2873	3303	1.31 (1.18 to 1.44)	
AKI stage				<0.001
<=1	2477	1576	1.63 (1.33 to 1.99)	
1~2	1636	2263	1.58 (1.40 to 1.79)	
3	4464	3570	1.19 (1.06 to 1.34)	
Potassium				0.637
<=4.5	6025	4785	1.43 (1.29 to 1.57)	
>4.5	2551	2624	1.40 (1.23 to 1.59)	
Anion gap				<0.001
<=16	6173	4129	1.64 (1.47 to 1.83)	
>16	2401	3280	1.17 (1.05 to 1.31)	
BUN				<0.001
<=20	5005	3099	1.59 (1.39 to 1.82)	
>20	3572	4309	1.31 (1.20 to 1.44)	
SCr				<0.001
<=1.2	6055	4158	1.56 (1.40 to 1.74)	
>1.2	2521	3251	1.24 (1.11 to 1.39)	
Malignancy				0.926
Diseased	589	1047	1.53 (1.27 to 1.83)	
Health	7988	6362	1.38 (1.26 to 1.51)	
Shock				<0.001
Diseased	946	1528	1.11 (0.97 to 1.28)	
Health	7631	5881	1.52 (1.38 to 1.67)	
Deficiency anemias				0.293
Diseased	4280	3817	1.47 (1.32 to 1.65)	
Health	4297	3592	1.35 (1.21 to 1.50)	
Sepsis				<0.001
Diseased	4746	5175	1.30 (1.19 to 1.42)	
Health	3831	2234	1.84 (1.55 to 2.19)	
Coronary artery disease				<0.001
Diseased	3736	2233	1.57 (1.36 to 1.81)	
Health	4841	5176	1.31 (1.19 to 1.44)	
Atrial fibrillation				0.002
Diseased	1405	1535	1.87 (1.52 to 2.30)	
Health	7172	5874	1.34 (1.23 to 1.46)	
COPD				0.094
Diseased	836	1024	1.27 (1.03 to 1.56)	
Health	7741	6385	1.44 (1.32 to 1.57)	
Vasoactive use				0.316
Unused	4238	3974	1.32 (1.17 to 1.49)	
Used	4339	3435	1.48 (1.33 to 1.64)	
RRT				0.179
Unused	8242	6895	1.42 (1.31 to 1.54)	
Used	335	514	1.36 (1.03 to 1.80)	

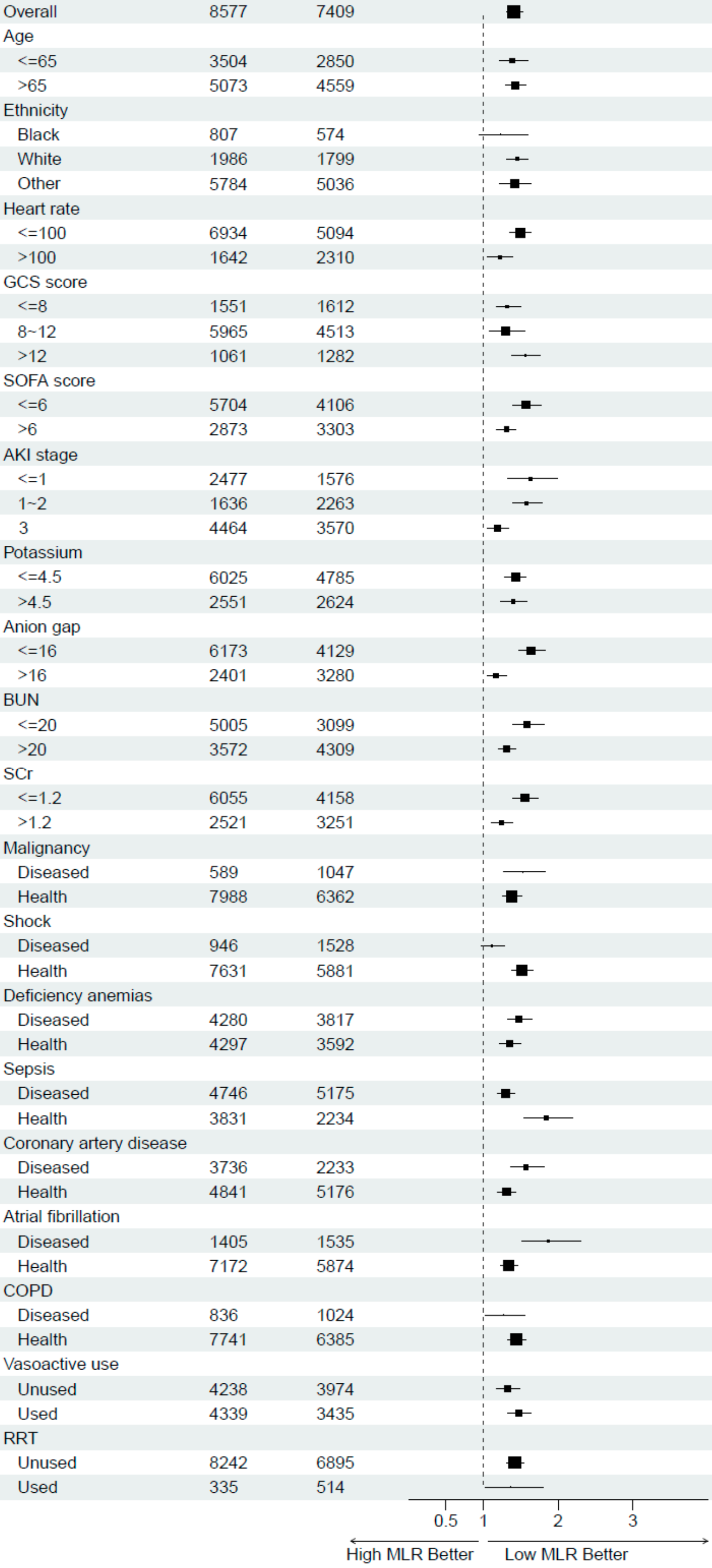

Figure S3. Subgroup analysis of the associations between 30-day all-cause mortality and the lymphocyte-to-lymphocyte ratio

Abbreviations: AKI Acute kidney injury, SCr serum creatinine, BUN Blood urea nitrogen, SOFA, GCS Glasgow Coma Scale, PO2 Partial pressure of oxygen, RDW red cell distribution width, MCV mean corpuscular volume, INR international normalized ratio, RRT renal replacement therapy, COPD chronic obstructive pulmonary disease.

HRs (95% CIs) were derived from Cox proportional hazards regression models. Covariates were adjusted as in model 2.

Table S1. Information of ROC curve in Figure S2.

**Variables**	**AUC**	**95% CI**	**Threshold**	**Sensitivity**	**Specificity**
MLR	0.64	0.63-0.65	0.48	0.627	0.587
Lymphocytes	0.61	0.60-0.62	1.20	0.627	0.548
Monocytes	0.55	0.54-0.57	0.63	0.475	0.617
Neutrophils	0.57	0.56-0.58	10.89	0.485	0.623
Platelets	0.52	0.51-0.54	282	0.261	0.798
SOFA score	0.68	0.67-0.69	7	0.606	0.663

AUC, area under the curve; CI, confidence interval; ROC, receiver operating characteristic.
